# Ferric Ions Modified Polyvinyl Alcohol for Enhanced Molecular Structure and Mechanical Performance

**DOI:** 10.3390/ma13061412

**Published:** 2020-03-20

**Authors:** Yu Su, Ying Wu, Ming Liu, Yan Qing, Jianbo Zhou, Yiqiang Wu

**Affiliations:** 1 College of Materials Science and Engineering, Central South University of Forestry and Technology, Changsha 410004, China; suyucsuft@163.com (Y.S.); wuying2000g@163.com (Y.W.); qingyan0429@163.com (Y.Q.); wuyiqiang@csuft.edu.cn (Y.W.); 2 Forestry New Technology Research Institute, Chinese Academy of Forestry, Beijing 100091, China

**Keywords:** polyvinyl alcohol, water-soluble polymers, ions modification, molecular structure

## Abstract

The highly crystallized molecular structure of polyvinyl alcohol (PVA) makes the polymer with poor performance in mechanical strength and water resistance. To modify the molecular structure of PVA and to diminish the complicated procedures and environmental impacts, ferric ions (in ion composite form) have been used to set the interactions with the molecule chains of PVA. The crystallinity, chemical groups change, and mechanical performance of the polymer has been confirmed by X-ray diffraction (XRD), Fourier transform infrared spectroscope (FTIR), and the bonding/membrane tensile strength test. The crystallinity of PVA is reduced from 41.6% to 7.7% with the addition of 2.0% of ferric ions. The tensile strength of the modified PVA membrane is increased by 240%. Moreover, with tougher structure and improved fluidity, the strength of ferric ions modified PVA bonded wood samples is increased by 157%. The modification of PVA with ion composite may have vast applications in many fields, such as paper industry, wood adhesives, functional materials, and polymer structure design.

## 1. Introduction

Polyvinyl alcohol (PVA) is one of the most important polymers used in the industry. It has the advantages of excellent performance in membrane formation [1,2], emulsifying [3], adhesives [4], and biocompatible materials [5]. It is a water-soluble polymer with controlled fluidity, which contains up to 99% of hydroxyls on its main molecule chain. The easy processing property, environmentally friendly nature, low cost, and abundant functional groups makes PVA a popular material in network establishment both in physically and chemically compounded materials, such as hydrogels [6,7,8]. PVA has excellent mobility, which is usually used to penetrate the surface of porous materials and to form smooth and stable bond lines. This makes it a common component in coatings and adhesives for paper and wood-based materials [9,10]. When used as an adhesive alone, the bonding strength of pure PVA is usually vulnerable to damage. This is caused by the large quantity of hydroxyls and the highly crystallized molecular structure in the polymer. To increase the bonding strength, PVA is usually used with other resins. For instance, PVA is widely used as a filler in formaldehyde-based and starch-based adhesives for the increase of their solid content and viscosity [11,12,13]. It is sometimes used as an emulsifier in isocyanate adhesives for even distribution or shelf time elongation purposes [14]. However, the addition of PVA in adhesives results in the decrease of water resistance of bonded materials due to the introduced large quantity of hydroxyls. This limits the use of PVA in indoor or dry conditions. To improve the water resistance and mechanical strength, it is recommended to block parts of hydroxyls in PVA molecule chains with additives or chemical modifications.

Thiamine has been used as a plasticizer to incorporate with PVA through a typical ultraviolet (UV) curing process [15]. By controlling the combination degree, the mechanical strength and water resistance of the PVA have been proved to be tunable. A physical–chemical PVA network structure with increased strength and stability was formed by adding dialdehyde nanocellulose in PVA solution [16]. However, the dispersion of the nanocellulose is a significant issue with the increase of PVA viscosity. The water sorption of PVA could be significantly decreased with the crosslinking effect between PVA molecule chains and glutaraldehyde [17]. The PVA structure obtained by chemical modification has been proven to be tough and stable. However, the chemical process is usually complicated and requires severe reaction conditions. Physical modification, such as the addition of nanoparticles, has also been widely studied. The tensile strength of PVA films could be enhanced with the addition of lignin nanoparticles [18]. Multi-layer membranes could also be prepared by depositing PVA and cellulose nanofibril layer by layer. The cellulose nanofibril layer inside PVA acts as enhanced layers in composites [19]. Physical modification needs no complicated process. However, the enhancement is mainly accounted for network entanglement or the hydrogen bond. Without chemical linkage, the structure of the material is not stable as compared to the chemical modified materials.

Ion composite has been widely used and reported as a modifier for polymers, which leads to enhanced properties of the modified material [20,21]. The structure of the pristine polymer is usually transformed with the addition of ion composite. This might attribute to the caution-induce, coordination effect, or chemical connections between the ions and the polymer matrix, which gives the compounded materials with special functions [22,23]. The modification of ion composite in polymer structures transformation has been extensively tried to meet special needs in many fields, such as elastomers [24], 3D printing [25], and hydrogels [26]. Elastomers obtain excellent mechanical properties and highly self-healing capability with proper ions induced molecular structure designs [27,28]. Self-healing abilities of coatings could also be realized with ionic interactions inside the polymer structures [29]. Compared to organic composites, ion composites are safer and more convenient for operation and utilization. However, it is difficult to predict the behavior of ion composites modified polymers. The lack of the theory also limits the molecular structure design based on ion composites modification.

Researches on the ion composites with catechol and carboxylic acid have been reported for their abilities as ligands. Theoretically, PVA is a polyol, which has the ability to crosslink with ion composites. Hydroxyls could also act as ligands under some conditions. However, the report of using ion composite to modify alcohols is rare. In this work, inspired by the Fe-catechol complex compound structure, ferric ions were introduced into PVA solution. The network structure of PVA was established by a simple catalyst-free and one-step co-heating process in water. The influence of ferric ions on PVA, such as the chemical groups, crystallinity, thermal property, mechanical performance, and water absorption has been explored and characterized. The results indicate that the modified PVA structure would be universal applicable in many fields such as hydrogel design, cartilage scaffold, elastomers, and membrane science.

## 2. Materials and Methods 

### 2.1. Materials

Polyvinyl alcohol, (C_2_H_4_O)_n_, in crystalline form, polymerization degree at 1750 ± 50, 99% pure, was purchased from Sinopharm Chemical Reagent Co. shanghai, China; Iron Ferric (III) chloride hexahydrate in crystalline form, 99% pure was purchased from Sigma-Aldrich Shanghai Trading Co., Ltd (Shanghai, China). The chemicals were used without further purification.

Poplar veneers with the dimensions of 200 mm × 80 mm × 2 mm, were purchased from the Yihua Timber Industry, Guangdong, China. Poplar strips were cut from the veneers to the dimension of 300 mm × 300 mm. All the wood strips were conditioned in a conditioning room at a temperature of 23 ± 2 °C and a relative humidity of 50% ± 5% for a week before further experiments.

### 2.2. Preparation of PVA Solutions and Bonded Wood Samples

Preparation of PVA solutions. The polymerized polyvinyl alcohol particles were dissolved in deionized water at the content of 8% on weight at 90 °C and stirred at 300 rpm for 2 h. Ferric chloride was added together with the PVA particles in different weight contents (based on the solution) of 0.0%, 0.6%, 1.0%, 1.4%, and 2.0% for the co-heating process. After that, the mixture was cooled down in a container at room temperature for at least one week before further tests. For simplicity, the sample is denoted as PVA-x%, while x is the weight content of FeCl_3_•H_2_O in the PVA sample. For example, the PVA sample containing 2.0% of ferric ions is denoted as PVA-2.0%.

Preparation of bonded wood samples. A three-layer plywood was prepared using a cold pressing process under 0.9 MPa for 2 h. The consumption of PVA solution was 20 ± 0.1 g per layer for all the samples. The PVA solution was brushed to the wood surface as uniformly as possible. In addition, to avoid the desiccation of the PVA solution on wood surface before the cold pressing process, the sample was required to be pressed once the brushing process was finished. Moreover, the wood veneers were stacked with the fiber direction perpendicular to each other for better mechanical strength of bonded wood samples. After the cold pressing process, all the samples were put in a conditioning room for a week at a temperature of 23 ± 2 °C and a relative humidity of 50% ± 5% before further tests.

### 2.3. Characterizations

Fourier transform infrared spectroscope (FTIR). The pure PVA and ferric ions containing PVA (PVA-2.0%) membranes were obtained through a drying process. In detail, the sample solution was poured into a glass-surface vessel and dried under room temperature for three days. The membranes were cut into the size of 5 mm × 5 mm for the characterization in an attenuated total reflection FTIR (ATR-FTIR, Nicolet iN10 MX FT-IR Microscope, Thermo Fisher Scientific, Waltham, MA, USA). The scanning range was from 650 to 4000 cm^−1^ in wave number. The scanning was repeated twice for the same membrane but in different locations for each sample.

X-ray diffraction (XRD). Membranes with various ferric ions contents were prepared using the same method in ATR-FTIR characterization. X-Ray diffraction patterns were collected on a D8 advance (Bruker Corporation, Billerica, MA, USA) X-ray diffractometer, operated at a rate of 5 degree/min and 2 theta degree from 10° to 90°. The data analysis was conducted by the MDI Jade 6 software (MDI, Materials Data, Inc., Livermore, CA, USA).

Differential scanning calorimetry (DSC). The PVA solutions with and without ferric ions were frozen dried at −60 °C for three days to get rid of moisture. Then, the samples were smashed into powders. Before smashing, the samples were immersed in liquid nitrogen for easy processing. About 5 mg of powder sample was used and sealed in the aluminum container for the test in a Q20 differential scanning calorimetry (DSC, TA instrument, New Castle, DE, USA). Before testing, the baseline was calibrated. The experiment was carried out from 30 to 300 °C with a temperature ramp rate of 10 °C/min.

Bonding strength. The bonding strength of the bonded wood specimens was measured according to a standard test method (GB/T 17657-2013). In detail, the bonded wood samples were cut into dimensions of 100 mm × 25 mm, which the wood fiber direction was parallel to the length of the specimen. Two notches were sawed on both sides of the specimen, which formed a central area with the size around (25 ± 1) mm × (25 ± 1) mm. The notch cut around 2/3 through the core of the specimen. The tensile shear strength of the specimens was measured using a WDW-10E universal mechanical experiment machine (Jinan Times Golden Testing Machine Co., Ltd, Jinan, China) at a loading speed of 10 mm/min. The force was recorded at the break load. The tensile shear strength was calculated using Equation (1) below. Seven specimens were tested for each PVA bonded wood sample.

(1) $$P=\frac{F_{max}}{b\times t}$$

where P is the bonding strength (MPa); *F_max_* is the break load (N); *b* and *t* are the width and the length of the bonding area (mm).

Membrane tensile strength. Membranes were made from the PVA solutions with ferric ions content of 0.0%, 0.6%, 1.0%, 1.4%, and 2.0%. Typically, PVA solutions were poured into culture dishes with 5 cm in diameter. The membranes were collected after drying under room temperature for three days. The weight of PVA solution was kept the same for the membrane preparation. The small culture dish was chosen to avoid uneven distribution or thickness of the formed membrane. The membranes were cut into a dumbbell shape with the dimension of 18 mm in length by using a model. The testing area is around 6 mm in length and 2 mm in width. The tensile strength was tested using a force meter with controlled movement components. The loading rate was controlled manually around 5 mm/min using a force meter. Three specimens were tested for each PVA sample. The tensile strength (σ) was calculated using Equation (2) as follows:

(2) $$\sigma =\frac{F}{b\times d}$$

where *F* is the load at break point (N); *b* and *d* are the width and the thickness of the membrane specimen (mm).

Microstructure observation. Scanning electron microscope (Quantum 450, FEI, Hillsboro, OR, USA) was used to observe the fracture surface of the PVA bonded wood samples. This aims to see the effect of ferric ions to the morphology of the PVA polymer.

Water absorption rate. The membrane specimens with relatively even thickness were prepared. In detail, 10 ± 0.1 g of the PVA solution was paved into a glass culture. The membrane was collected after drying under room temperature for three days. Then, the membrane specimens were stacked and cut into the same shape to assure the same surface area. Later, the membranes were immersed into a 100 mL beaker with 50 mL of water to avoid the side effects, such as the pressure of water. Finally, the weight change was recorded at the time of 0, 1, 2, 3, 5, 10, 15, 20, 30, 45, 60, 90, and 120 min.

## 3. Results and Discussion

### 3.1. Chemical Composition and Shecmatical Mechanism of Ferric Ions Modified PVA

#### 3.1.1. Chemical Groups of Dried PVA Solutions

**Figure 1:**
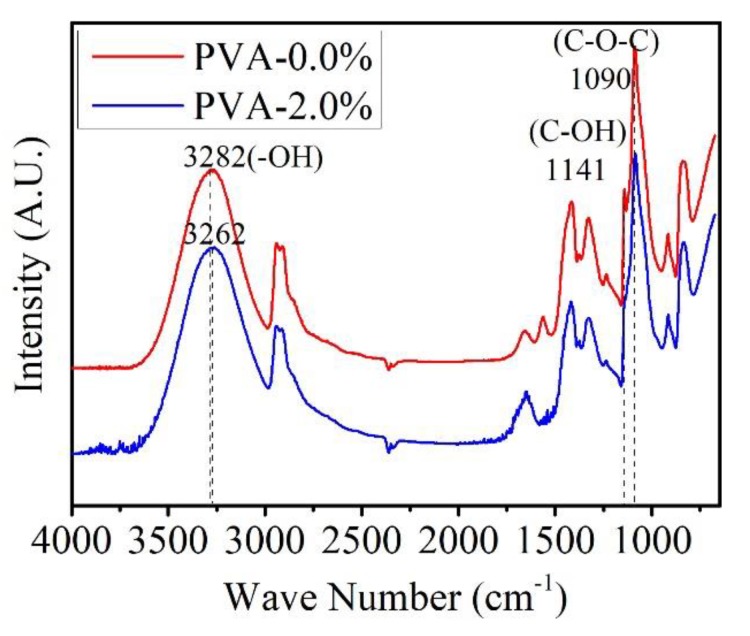
ATR-FTIR spectra of membranes of pure polyvinyl alcohol (PVA) and PVA with 2.0% of ferric ions.

ATR-FTIR experiments were used to investigate the influence of ferric ions to the molecule chains of PVA. As shown in Figure 1, a strong band corresponding to the hydrogen-bonded hydroxyl is observed at 3282 cm^−1^ in the sample of PVA-2.0%. In the spectrum of PVA-0.0%, this absorption band is shifted to 3262 cm^−1^. The shift of the absorption peak is caused by the bond energy change, which might attribute to the coordination behavior of Fe (III) [30,31]. Ferric ion is a high spin d5 species and generally forms hexacoordinate complexes with suitable ligands. Ferric ions would be crosslinking with hydroxyls as it is to ligands. In the coordination process, Cl– was replaced by –OH to form FeO(OH). Stretching absorption of Fe-O-C bond is supposed to appear at 1090 cm^−1^ [32]. However, it is overlapped by the C-O-C bond in this study. While compared to the other peaks, such as the C-H stretching vibration at 2941 and 2910 cm^−1^, the peaks are rarely shifted. It indicates that the results and the combination effects are reliable. In PVA-0.0%, there is a sharp peak at 1141 cm^−1^, which is corresponding to the stretching absorption of C-OH. However, the peak of PVA-2.0% at this position is ambiguous. This is probably caused by the ion cation effect on hydroxyl groups. The results indicate that the addition of Ferric ions in PVA might enhance the connections of the polymer molecule chains through possible improved bond energy and ion cation effects.

#### 3.1.2. Crystallinity of Dried PVA Powder

**Figure 2:**
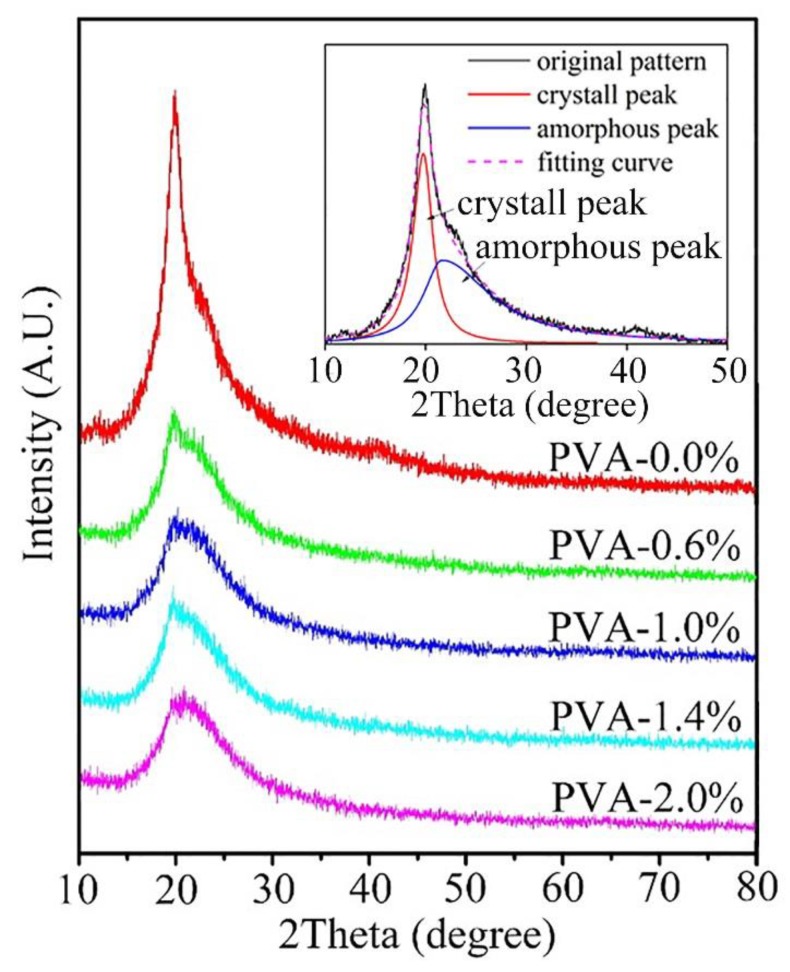
XRD curves of PVA powder containing 0.0%, 0.6%, 1.0%, 1.4%, and 2.0% of ferric ions (the inset is a typical fitting and simulation process of the crystalline fraction calculation).

The X-ray diffraction was used to investigate the influence of ferric ions to the crystallinity of PVA. As shown in Figure 2, the pristine PVA sample has a strong signal and sharp peak at 2θ = 19.8°. It has been pointed out in many researches that the signal at 2θ = 19.8° is a typical and significant diffraction peak of PVA [33]. To quantify the change of crystallinity, it is better to calculate the crystalline fraction of the PVA samples. This calculation requires the extraction of crystalline diffraction area from the original XRD pattern. The MDI Jade 6 software was used to run the fitting and simulation process. In detail, the amorphous peak of the XRD pattern was generated with an automatic full spectrum fitting process at first. As shown in the inset of Figure 2, the crystal peak was fixed at 2θ = 19.8°. The amorphous one was fixed at 2θ = 21.6° [33]. Second, the spectrum was refined with an automatic calibration process. The results were reported after the minimum residual error of fit was obtained in the software. The reported crystalline fraction from the fitting process is shown in Table 1. Compared to the pristine PVA sample, the crystallinity of the polymer is decreased with the addition of ferric ions. The increase amount of ferric ions addition also results in further decrease of crystallinity in PVA. This might attribute to the ion effects inside the molecular structure of PVA samples. The dominated hydrogen bonding crystallization process in the pristine PVA was prevented by the introduction of ions. The change of crystalline fraction in PVA is tunable with the content control of ferric ions.

#### 3.1.3. Thermal Properties of PVA Samples

**Figure 3:**
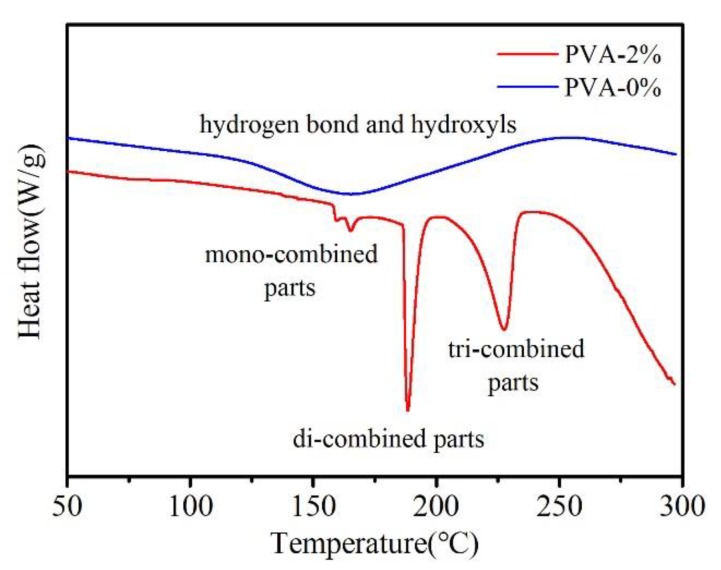
DSC curves of freeze-dried pure PVA and PVA containing 2.0% of ferric ions.

To investigate the combination state of ferric ions in PVA solution, freeze-dried samples of PVA-0.0% and PVA-2.0% were used for the DSC characterization. As shown in Figure 3, there is only one smooth and wide peak signal from 120 to 250 °C in PVA-0.0%. The melting temperature of PVA is around 240 °C. The weight loss under the melting temperature is attributed to the dehydration from hydroxyls [34,35]. This is also how the hydroxyls is consumed in the pure PVA sample. In comparison, the smooth peak is divided into several peaks in PVA-2.0%. As reported in other works, ferric ions could coordinate with hydroxyls in PVA. Each ion could coordinate three hydroxyls at most [30,36,37]. The first and the second peak are very close to each other both in temperature and in heat value. This is the signal of rare residue hydroxyls and mono-combined ferric ions in PVA, which is also in minority. Two strong signals arise at 188 and 228 °C, which might represent the destruction of di-combined ferric ions and tri-combined ferric ions, respectively. With the increased combination degree, the heat value for the destruction is also increased deeply. It illustrates that the highly combined structure is more stable than the partly combined ones. Thus, the tri-combined form is the main form for ferric ions to construct the most stable structure.

#### 3.1.4. Schematic Mechanism of Ferric Ion Modification Effects on PVA

**Figure 4:**
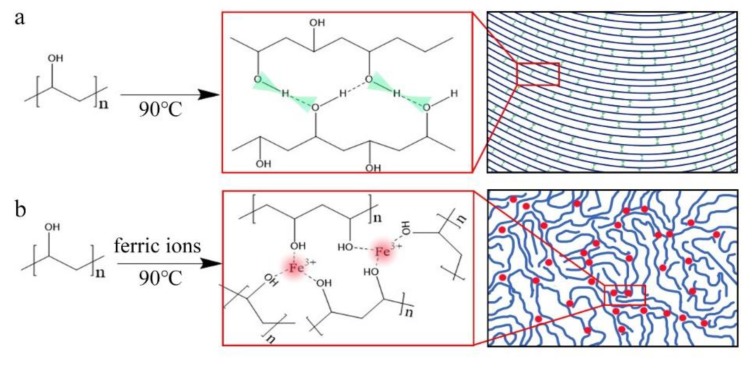
Schematic illustration for the structure formation of (**a**) pure PVA and (**b**) ferric ions modified PVA.

The mechanism of the ferric ion modified PVA could be assumed based on the above analysis. In the drying process of pure PVA solution, ordered arrangement of molecule chains is obtained due to the strong intermolecular hydrogen bond, as shown in Figure 4a. The highly sequential structure results in a highly crystallized structure. In the thermo analysis, the hydroxyls are supposed to be dehydrated and to form ether bonds during the heating process. This results in a smooth and wide peak in Figure 3. Ferric ions have an influence on the formation of intermolecular hydrogen bond. The shift in ATR-FTIR spectrum indicates that the combination between ferric ions and hydroxyls is established. In the drying process, the ferric ions could capture hydroxyls, which prevents the formation of a strong hydrogen bond. Thus, the crystallinity is decreased sharply with the addition of ferric ions, as shown in Table 1. This could also be found in the amorphous structure, as shown in Figure 4b. In the thermo analysis, the wide and smooth peak is divided into three sharp peaks, indicating that the combination is not the same. The tri-combined structure is the main form as obtained from the heat values.

### 3.2. Mechanical and Swelling Behavior of PVA with and without Ferric Ions Modification

The mechanical properties of PVA and ferric ions containing PVA were evaluated by using the composites as adhesives to bond wood. The modified PVA has proper viscosity and mobility. This meets the requirements for forming a good wood bonding structure since wood is a porous material. From the last part in the above section, it indicates that the molecular structure might be transformed by the addition of ferric ions. It is assumed that the transformation in molecular structure results in a better mechanical strength. To further explore the ferric ions effect, the morphology of the fracture surface of the bonded wood sample was observed.

In addition, tensile strength of PVA membranes was tested to reveal the connection between mechanical strength and crystallinity change in the polymer structure. The swelling behavior of the membrane was also studied to investigate the change of sensitivity to water.

#### 3.2.1. Bonding Performance of PVA Bonded Wood Samples

**Figure 5:**
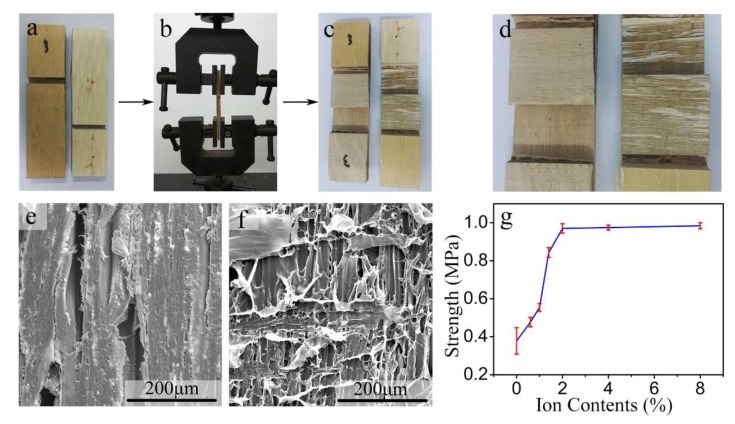
(**a**) Tailored bonded wood specimen; (**b**) set on the universal mechanical testing machine; (**c**) break sample of a; (**d**) the fracture surface of PVA-0.0% (left) and PVA-2.0% (right) specimen; SEM images of (**e**) PVA-0.0% and (**f**) PVA-2.0% specimen; (**g**) bonding strength of the bonded wood samples with different addition amounts of ferric ions.

To explore the effect of ferric ions to the adhesion strength of the modified polymers, PVA solution with different contents of ferric ions were prepared and used to bond wood veneers. The specimens were prepared and tested, as shown in Figure 5a–c.

As shown in Figure 5g, the bonding strength of the PVA is increased with the addition of ferric ions. Without ferric ions, the shear strength of the bonded wood samples is around 0.38 MPa. While the bonding strength of PVA-2.0% sample is around 0.97 MPa. With further addition amounts of ferric ions, the bonding strength has been improved slightly. The result is opposite to the regularity of crystallinity. The bonding strength is decreased with the increase of crystallinity. To study the mechanism, the fracture surface of PVA-0.0% and PVA-2.0% specimens was observed, as shown in Figure 5d–f. 

In the PVA-0.0% specimen, the fracture surface is quite flat. The destruction of wood has not been observed. In the amplified sample of PVA-0.0% in Figure 5e, the surface is covered completely by the polymer. The wood fiber structure is also ambiguous. It might be the break of the interface between PVA and the wood, which has caused a relatively weak bonding strength. The adhesion strength is mainly attributed to the hydrogen bond between PVA and the wood surface. However, in the PVA-2.0% specimen, the wood has been partly destroyed, as shown in Figure 5d. With the magnification in Figure 5f, a rough surface and the wood fiber structure can be clearly seen. This means that the ferric ions modified PVA has a great dispersion or penetration in wood structures. In comparison, the destruction of the polymer can be observed, which might take place in the interior polymer structure rather than the PVA-wood interface. The strong bonding strength might account to the combination effect between ferric ions and PVA molecule chains.

**Figure 6:**
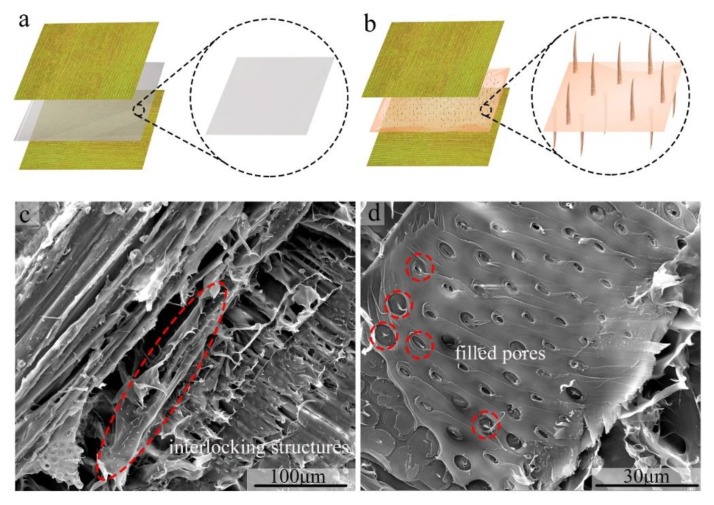
Models for the internal structure of PVA samples in bonding wood: (**a**) The pure PVA forms flat structures (**b**) ferric ions modified PVA forms interlocking structure; SEM images of (**c**) numerous needle-like interlocking structure formed by filling wood porous structure captured in PVA-2.0% sample; (**d**) the modified PVA filled pores in wood structure.

Wood absorbs water readily. Water would be absorbed rapidly when the PVA solution is brushed on wood veneer. PVA molecule chains could be rapidly arranged by the intermolecular hydrogen bonds without water. This forms flat PVA layers, as shown in Figure 6a, which makes it difficult to form interlocking structure in the bond line. The fracture takes place at the interface section rather than the polymer layer during the mechanical testing. After the addition of ferric ions, most of the hydroxyls are occupied. The movement of PVA molecule chains are no longer limited by the strong hydrogen bonds. This leads to a great fluidity of the polymer, which could penetrate deeper into the wood porous structure during the cold pressing process. Once the polymer is dried, interlocking structures could be achieved, as shown in Figure 6b. To support the hypothesis, digital images were taken for the fracture surface. As shown in Figure 6c, the PVA-2.0% specimen shows an extensively needle-like structure. They consist of the sequence of the pore arrangement in wood structure. In Figure 6d, the pore structure is filled with the modified PVA, which forms the needle-like structure. They are the residues of the broken interlocking structure, which supports our assumption. The similar structure has not been observed in pure PVA bonded wood samples.

#### 3.2.2. Mechanical and Swelling Behavior of PVA Membranes

**Figure 7:**
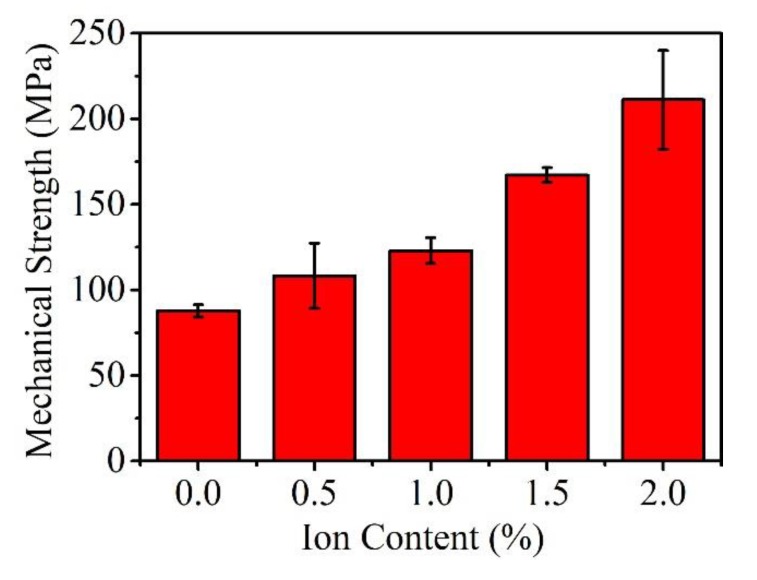
Membrane tensile strength of pure PVA and modified PVA samples with various contents of ferric ions.

The tensile strength of PVA membranes is shown in Figure 7. Without ferric ions, the tensile strength is around 87.7 MPa, which is the lowest value among all the samples. With the increased amount of ferric ions, the membrane strength is increased to a maximum value at 211.1 MPa. This is consistent with the tensile shear strength of PVA bonded wood samples. It indicates that the combination between ferric ions and hydroxyls is stronger than the hydrogen bond itself. The bonding strength in bonded wood samples was increased with this stronger combination. The interlocking structure might not be the only factor that affects the bonding strength. Ferric ions could improve the mechanical performance of PVA, which makes it harder to be broken under pressure. This also corresponds to the fact that ferric ions affect the crystallinity of PVA. The destruction of the bonded wood samples occurs at the polymer itself rather than the interfaces between wood and PVA layer.

**Figure 8:**
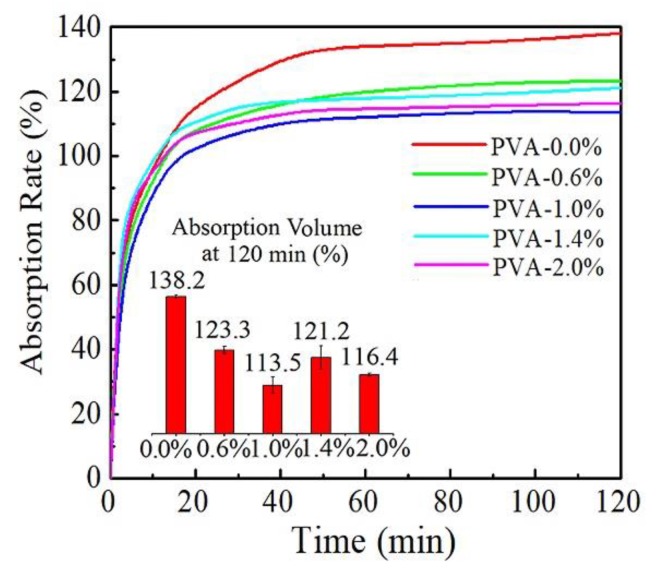
Swelling behavior of pure PVA membranes and PVA membranes with various contents of ferric ions.

The swelling behavior of the membranes was also tested to evaluate the change of their sensitivity to water. There is a significant difference in maximum water absorption volume between the PVA-0.0% sample and ferric ions containing samples. However, there is no significant difference among samples in the absorption rate. This is consistent with the regularity of crystallinity change. In Figure 8, an equilibrium state is considered when the change of the absorption rate is lower than 0.5% per minute. For each curve, three regions could be divided: Linear increase region, stable increase region, and equilibrium region, which are decided by the gradient. In the pristine PVA sample, the three regions are obvious. With the increased amount of ferric ions addition, the second region begins to narrow down. It is ambiguous to see the border between the stable increase region and the equilibrium region in sample PVA-2.0%. The first stage is considered as an absorption process of the hydroxyls on the surface. Thus, it appears to be a linear increase. The second stage is the dispersion of moisture from the surface to the inside structure. A faster water dispersion rate suggests that the interior structure is easier to be penetrated. Compared to PVA-0.0%, the hydroxyls in ferric ions modified PVA molecular structure are partly blocked. Thus, the addition of ferric ions decreases the time for water dispersion process in swelling. The third stage occurs because of the limitation of the monolithic construction. The ferric ions could block part of the hydroxyls in PVA membranes and shorten the time for reaching an equilibrium state of water absorption. However, the hydrophilic properties of PVA has not been changed significantly.

## 4. Conclusions

Ferric ions showed a significant influence on the arrangement of PVA molecule chains in this study. Ferric ions modified PVA produced by the catalyst-free, one-step co-heating method has the potential to be used in polymers for performance improvement, including mechanical strength, fluidity, penetrating properties, and water resistance. With the modification of ferric ions, the hydrogen bond inside PVA molecule chains could be interfered, which results in the weakened crystallization process of PVA. This behavior improves the fluidity or penetration property of PVA towards porous materials. The bonding strength of PVA to wood veneer is increased by 157%. The mechanical strength of the modified polymer membrane is improved by 240%. The water resistance of PVA is also improved with the introducing of ferric ions since the hydroxyls could be partly blocked. This feature has the potential to be used for enhancing waterproof performance of polymers. The effect of ferric ions to PVA provides us with an insight in the polymer structure or function design. PVA film is one of the most important membrane formation polymers used in the industry. The quick desiccation of the PVA solution might be also influenced or slowed by the addition of ferric ions, which leads to an even distribution or thickness of the polymer.

## Figures and Tables

**Table 1:** Crystalline fraction of PVA samples calculated from the XRD curves.

Sample	Crystalline Fraction
PVA-0.0%	41.6%
PVA-0.6%	26.9%
PVA-1.0%	20.2%
PVA-1.4%	14.6%
PVA-2.0%	7.7%
